# The association of burnout with occupational stressors among physicians and medical technicians/nurses in the Federation of Bosnia and Herzegovina: a cross-sectional study

**DOI:** 10.3325/cmj.2026.67.85

**Published:** 2026-04

**Authors:** Ankica Mijić Marić, Marnela Palameta, Mirela Mabić, Amra Zalihić, Edita Černy Obrdalj, Anđela Pupić, Marina Berberović, Sandra Kostić

**Affiliations:** 1 Department of Family Medicine, School of Medicine, University of Mostar, Health Care Center Mostar, Mostar, Bosnia and Herzegovina; 2 Faculty of Economics, University of Mostar, Mostar, Bosnia and Herzegovina; 3 Center of Urgent Medicine, University Clinical Hospital Mostar, Mostar, Bosnia and Herzegovina; 4 Department of Anatomy, Histology and Embryology, University of Split, School of Medicine, Split, Croatia

## Abstract

**Aim:**

To assess the prevalence of burnout syndrome and its association with occupational stressors among physicians and medical technicians/nurses in the Federation of Bosnia and Herzegovina (FBiH).

**Methods:**

This cross-sectional study was conducted between February 12 and April 12, 2025, using an anonymous online survey. Structured self-administered questionnaire scales were used to assess burnout (Copenhagen Burnout Inventory) and workplace stressors (Occupational Stress Questionnaire). The association of personal, work-related, and patient-related burnout with occupational stressors was assessed. The survey was sent to the members of the Union of Physicians and Dentists in the FBIH, who were asked to forward the link to their medical technicians and nurses.

**Results:**

Overall burnout symptoms were reported by more than half of the respondents (53.0%), with 75.7% experiencing at least one form of burnout. Personal burnout was the most prevalent (70.9%), followed by work-related burnout (54.2%) and patient-related burnout (40.1%). In multiple regression analysis, overall burnout was driven by the cumulative impact of multiple stressors rather than by any single factor. All stressors were positively correlated with burnout, but conflicts and communication at work (for personal and work burnout) and public criticism (for patient-related burnout) stood out as the strongest predictors.

**Conclusion:**

This study observed a high prevalence of burnout among physicians and medical technicians/nurses in the FBiH. All examined domains of professional stressors were correlated with all burnout dimensions. Given the importance of preventing and alleviating burnout among health care professionals, this research provides insight into key factors contributing to burnout.

Burnout syndrome (BOS) is a psychological phenomenon resulting from chronic workplace stress that has not been successfully managed (1). In people-oriented professions, particularly health care, burnout has long been recognized as an important occupational risk (2). BOS has been linked to patient safety issues and a decline in the quality of provided care (3). Furthermore, it undermines the sustainability of health care organizations by driving employees to disengage professionally and leave the workforce, which ultimately lowers the overall standard of patient care (4).

Research has identified numerous organizational risk factors associated with burnout. Six key domains within the organizational structure contribute to this syndrome: work overload, lack of control, insufficient recognition and reward (whether financial, institutional, or social), breakdown of community and job-related relationships, a lack of fairness, and conflicting values (2,5). Beyond professional performance, chronic occupational stress is a known precursor to mental and behavioral disorders, including anxiety, depression, and alcohol dependence, as well as physical morbidities such as cardiovascular diseases (6-9).

Although the prevalence of burnout varies significantly across different health care professions and settings, burnout rates had reached crisis proportions before the COVID-19 pandemic — affecting nearly 30% of nurses (10,11) and over 40% of physicians (12-15). The pandemic’s extreme demands triggered a sharp escalation, with rates often soaring above 70% in some settings (16-21). Although these figures have slightly declined since the pandemic's peak, they remain significantly higher than pre-pandemic levels (22). Our study during the pandemic period showed that 77% of health care workers in the Federation of Bosnia and Herzegovina experienced some form of burnout (23).

Despite the high prevalence of burnout reported during the pandemic, there is a lack of data on how these rates have evolved in the post-pandemic period within the specific socio-economic context of the FBiH. Furthermore, the relative contribution of different occupational stressors to burnout among physicians and medical technicians in this region remains insufficiently explored. Therefore, this study aimed to determine the prevalence of burnout among physicians and medical technicians/nurses in FBiH after the pandemic, and to identify specific occupational stressors most strongly associated with the syndrome.

## Respondents and methods

### Study design

This cross-sectional study was conducted between February 12 and April 12, 2025, using an online survey based on the Copenhagen Burnout Inventory (CBI) and Occupational Stress Questionnaire (OSQ). Data were collected via Google Forms. An invitation to participate in the online survey was sent to the members of the Union of Physicians and Dentists in FBiH, who were asked to forward the link to their medical technicians and nurses (snowball sampling technique). The consent to use the communication channels of the Union was obtained previously (number: 01-1-1/2025, date: January 31, 2025). The online survey also included an introductory note stating the study's aim. Research participation was voluntary and anonymous. The study included physicians and medical technicians/nurses from the FBiH, regardless of age, health care status, job title, or employment status. The invitation to participate was resent after one month. Ethical approval was obtained from the Committee for Medical Ethics of the Faculty of Medicine, University of Mostar (01-I-2111/23).

### Instrument/measurement

The questionnaire consisted of three parts: basic sociodemographic questionnaire, the CBI, and the OSQ. The sociodemographic questionnaire collected demographic and work-related data: sex, age, the number of children, education, job profile, and work environment.

The CBI was developed by the National Institute of Occupational Health in Denmark. This validated psychometric instrument was designed to measure burnout through the core concepts of fatigue and exhaustion. The CBI consists of 19 specific burnout questions divided into three sections: personal burnout (general symptoms of exhaustion, 6 items); work-related burnout (7 items); and client-related burnout (6 items). The inventory was adapted by replacing the word “client” with the word “patient,” given our target population. Burnout was defined as a CBI score >50; a higher score represents a higher level of burnout (score 50 to 74 – moderate, 75 to 99 – high, and 100 – severe burnout) (24-26).

The standardized abridged form of the OSQ was used to examine the impact of the work environment on the experience of stress. This questionnaire was developed by the Finnish Institute of Occupational Health and has been in use since 1992 (27). The OSQ has been adapted and validated into the Croatian language (16,28). Respondents were offered 37 work stressors divided into six domains: work organization and financial issues (11 items), public criticism (6 items), dangers and harmfulness at work (5 items), conflict and communication at work (5 items), shift work (5 items), and professional and intellectual demands (4 items). Each item was rated on a five-point Likert scale (1 = “not at all stressful”; 5 = “extremely stressful”), with higher total scores reflecting greater levels of occupational stress.

### Validity and reliability

Forward and backward translation and preliminary pilot testing of the CBI questionnaire were conducted in our previous study (23). The Kaiser-Meyer-Olkin measure of the sampling adequacy was 0.958, and the Bartlett’s test of sphericity was statistically significant (*P* < 0.001). The three factors explained 68.073% of the variance: factor 1 explained 53.505%, factor 2 explained 10.605%, and factor 3 explained 3.964% of the variance. Cronbach’s alpha for the whole scale was 0.950, and the interclass correlation was 0.950 (*P* < 0.001). Cronbach’s alpha for individual dimensions/scales was the following: personal burnout scale 0.896, work-related burnout scale 0.904, and patient-related burnout scale 0.908.

Cronbach’s alpha for the OSQ ranged from 0.899 to 0.954, indicating a high level of internal reliability of the used scales. Average stressor values ranged from 2.26 to 2.77. The distribution of the results was within the limits of normality, with skewness from 0.286 to 0.787 and kurtosis from −1.216 to −0.323. The values of the asymmetry indicator were positive, which indicated a slight shift in the distribution toward lower values of the scale, ie, that a larger number of respondents assessed the intensity of the stressor with lower scores.

### Statistical analysis

The Kolmogorov-Smirnov test was employed to assess the normality of data distribution. Categorical variables are expressed as frequencies and percentages, whereas numerical variables are expressed as mean (M) and standard deviation (SD). The independent-samples *t* test and one-way analysis of variance (ANOVA) were used to assess group differences in workplace and sociodemographic factors. The correlation between the stressors and burnout aspects was evaluated using the Pearson correlation coefficient (r). A series of multiple regression analyses was performed to assess the predictive significance of stressors in explaining the variance across levels of burnout. The significance level was set at α = 0.05. Statistical analysis was conducted using SPSS Statistics for Windows, version 25.0 (IBM Corp., Armonk, NY, USA).

## Results

### Respondents’ characteristics

Overall, 670 respondents from nine out of ten counties in the FBiH participated in the survey. At the time of the survey, the Union of Physicians and Dentists in FBiH had 4670 members (29), which is over 80% of the total number in FBiH (30). The response rate was 11.2% (523/4670). The snowball sampling technique prevented us from calculating the response rate for medical technicians/nurses. Most of the respondents were women, aged up to 50 years, with up to 20 years of work experience, a college degree, married, physicians, and specialists (Table 1).

**Table 1:** Characteristics of respondents (N = 670)

		Number of respondents	%
Sex	male	139	20.7
	female	531	79.3
Age (years)	35 or less	256	38.3
	36-50	303	45.3
	51 or more	110	16.4
Married	no	218	32.5
	yes	452	67.5
Children	yes	254	37.9
	no	416	62.1
Work experience (years)	10 or less	282	42.1
	11-20	251	37.5
	21 or more	137	20.4
Education	high school	84	12.5
	college	479	71.5
	MSc or PhD	107	16.0
Level of health care	primary care	326	48.7
	secondary/tertiary care	344	51.3
Qualification	physicians	523	78.1
	medical technicians/nurses	147	21.9
Specialization (physicians)	specialists	339	64.9
	residents	116	22.2
	no specialization	67	12.8

### Burnout

Burnout symptoms were reported by 53.0% of the respondents (n = 355), with no respondent scoring 100. The mean overall score and the scores on dimensions ranged from 40 to 60. The highest mean was achieved on the personal burnout domain, and the lowest score on patient-related burnout (Table 2).

**Table2:** The level of burnout, in general and by dimensions

	Total burnout	Personal burnout	Work-related burnout	Patient-related burnout
Min-max	8-96	8-100	0-100	0-100
Mean (standard deviation)	51.0 (17.6)	58.1 (17.8)	51.2 (20.4)	43.8 (20.7)
No burnout, n (%)	315 (47.0)	195 (29.1)	307 (45.8)	401 (59.9)
Moderate, n (%)	287 (42.8)	323 (48.2)	262 (39.1)	204 (30.4)
High, n (%)	68 (10.1)	141 (21.0)	99 (14.8)	57 (8.5)
Severe, n (%)		11 (1.6)	2 (0.3)	8 (1.2)
Result 0, n (%)	0	0	1	11
Result 100, n (%)	0	11	2	8

Personal burnout was experienced by 70.9% of respondents (n = 457), work-related burnout by 54.2% (n = 363), and patient-related burnout by 40.1% (n = 269) (Table 2). Overall, 75.7% of participants experienced some form of burnout (a score higher than 50% in at least one dimension of burnout). A total of 18.5% of participants experienced only one form of burnout, 24.8% two forms of burnout, and 32.4% all three forms of burnout.

Female respondents reported higher burnout in the personal burnout domain. Higher patient-related burnout was reported by respondents working in primary health care. Among physicians, specialists reported higher total and work-related burnout than residents, and physicians without a specialization reported higher patient-related burnout than both residents and specialists (Table 3).

**Table 3:** The level of burnout at work in regard to the demographic and work characteristics of respondents

		Total burnout		Personal burnout		Work-related burnout		Patient-related burnout	
		M*	SD*	M	SD	M	SD	M	SD
Sex	male	50.5	19.2	54.4	19.0	50.3	21.2	46.7	22.4
	female	51.2	17.1	59.1	17.4	51.4	20.2	43.1	20.2
	p^†^	0.664		0.006		0.561		0.064	
Age (years)	35 or less	50.9	17.0	58.2	17.1	50.6	20.5	44.0	20.3
	36-50	51.8	17.8	58.8	18.2	52.4	20.3	44.2	20.9
	51 or more	49.0	18.2	55.8	18.4	49.0	20.3	42.2	21.0
	p^‡^	0.348		0.327		0.262		0.678	
Work experience (years)	10 or less	50.7	16.9	57.9	17.1	50.4	20.3	43.9	20.2
	11-20	51.7	17.7	58.6	18.1	52.0	20.0	44.5	20.6
	21 or more	50.4	18.5	57.5	18.9	51.2	21.3	42.4	21.8
	p^‡^	0.722		0.813		0.650		0.647	
Level of health care	primary care	52.2	17.7	58.5	17.9	51.1	20.4	47.1	20.1
	secondary/tertiary care	50.0	17.4	57.7	17.8	51.2	20.4	40.7	20.9
	p^†^	0.102		0.570		0.951		<0.001	
Qualification	physicians	51.0	17.3	57.6	17.4	51.3	20.1	44.2	20.6
	medical technicians/nurses	51.1	18.4	60.0	19.3	50.7	21.3	42.6	21.1
	p^†^	0.985		0.151		0.738		0.423	
Specia- lization	specialists	50.8	17.5	57.2	17.8	51.5	20.1	43.5	20.6
	residents	48.8	17.6	56.1	16.6	48.0	20.7	42.2	21.1
	no specialization	56.0	15.0	62.0	16.1	55.4	17.9	50.6	18.6
	p^†^	0.023		0.069		0.049		0.020	

*M – mean; SD – standard deviation.

†t-test for independent samples.

‡one-way ANOVA for independent samples.

### Occupational stressors

Table 4 shows the descriptive parameters for the six investigated factors of stressors at the workplace. In the workplace organization and financial issues domain, the most important stressors were insufficient number of employees (M = 3.06) and administrative burdens (M = 3.04), followed by poor work organization (M = 2.99), work overload (M = 2.96), and deadline pressure (M = 2.66). In the public criticism domain, the most important stressor was misinformed patients due to media and other sources (M = 2.86), while the least important stressor was conflicts with patients or family members (M = 2.32).

**Table 4:** Descriptive statistics for workplace stressors domains

	Stressor domains	Cronbach alpha	Mean	Standard deviation	Skewness	Kurtosis
1	Workplace organization and financial Issues	0.954	2.77	1.22	0.286	−1.216
2	Public criticism	0.939	2.45	1.27	0.620	−0.879
3	Dangers and harmfulness at work	0.954	2.29	1.18	0.787	−0.323
4	Conflict and communication at work	0.931	2.26	1.24	0.787	−0.538
5	Shift work	0.913	2.58	1.32	0.401	−1.131
6	Professional and intellectual demands	0.899	2.41	1.21	0.658	−0.668

Regarding dangers and harmfulness at work, the most important stressor was caring for patients with incurable illnesses (M = 2.72), while the least important stressors were fear of needle-stick injuries (M = 2.24) and the risk of infection (M = 2.30).

In the conflict and communication at work domain, the most important stressor was poor communication with superiors (M = 2.69), while the least important stressors were conflicts and poor communication with colleagues or coworkers (M = 2.21–2.32).

In the shift work domain, the most important stressors were 24-hour shifts (M = 2.97), 24-hour responsibility (M = 2.90), and night shifts (M = 2.88), whereas the least important stressor was standard shift work (M = 2.39). Finally, in the professional and intellectual demands domain, the leading stressor was the lack of continuing education (M = 2.81). The least important stressors were the introduction of new technologies (M = 2.20) and new professional information overload (M = 2.28) (Supplemental Table 1(Supplementary Table 1)).

Older respondents reported lower levels of stress across all investigated domains. Regarding the length of service, respondents with 21 or more years of experience reported significantly lower levels of stress in the domains of public criticism and professional demands. Employees working in secondary/tertiary level of health care reported significantly higher stress levels in conflicts and communication at work, shift work, and hazards and harms in work domains than those employed in primary care. Nurses and technicians reported higher stress associated with hazards and harms at work, whereas physicians reported higher stress levels associated with shift work.

Among physicians, specialists reported significantly lower levels of stress in four domains: workplace organization, public criticism, occupational hazards, and professional demands compared with residents. Specialists also reported lower stress related to workplace organization and financial issues than respondents without specialization (Table 5).

**Table 5:** Differences in workplace stressor domains by demographic and work characteristics

		Workplace organization and financial issues		Public criticism		Dangers and harmfulness at work		Conflict and communication at work		Shift work		Professional and intellectual demands	
		M*	SD*	M	SD	M	SD	M	SD	M	SD	M	SD
Sex	male	2.8	1.2	2.5	1.3	2.2	1.2	2.2	1.3	2.5	1.3	2.3	1.1
	female	2.8	1.2	2.4	1.3	2.3	1.2	2.3	1.2	2.6	1.3	2.4	1.2
	p^†^	0.869		0.936		0.551		0.682		0.291		0.353	
Age (years)	35 or less	2.9	1.2	2.6	1.3	2.4	1.3	2.4	1.3	2.6	1.3	2.5	1.2
	36-50	2.8	1.3	2.5	1.3	2.3	1.2	2.3	1.2	2.7	1.3	2.4	1.2
	51 or more	2.4	1.1	2.0	1.1	2.0	1.0	2.0	1.0	2.3	1.3	2.1	1.1
	p^‡^	0.005		<0.001		0.007		0.035		0.022		0.007	
Work experience (years)	10 or less	2.9	1.2	2.6	1.3	2.4	1.2	2.3	1.3	2.6	1.3	2.5	1.2
	11-20	2.7	1.3	2.4	1.3	2.3	1.2	2.3	1.2	2.6	1.3	2.4	1.2
	21 or more	2.6	1.2	2.2	1.2	2.1	1.0	2.1	1.1	2.5	1.4	2.2	1.2
	p^‡^	0.086		0.012		0.116		0.169		0.477		0.050	
Level of health care	primary care	2.7	1.2	2.4	1.3	2.2	1.1	2.0	1.1	2.4	1.3	2.3	1.2
	secondary/tertiary care	2.8	1.2	2.5	1.3	2.4	1.2	2.5	1.3	2.8	1.3	2.5	1.2
	p^†^	0.391		0.273		0.011		<0.001		<0.001		0.075	
Qualification	physicians	2.8	1.2	2.5	1.3	2.2	1.2	2.2	1.2	2.6	1.3	2.4	1.2
	medical technicians/nurses	2.7	1.3	2.4	1.4	2.5	1.3	2.3	1.3	2.3	1.3	2.5	1.4
	p^†^	0.698		0.702		0.044		0.384		0.016		0.596	
Specia- lization	specialists	2.6	1.2	2.3	1.2	2.1	1.1	2.2	1.2	2.6	1.3	2.3	1.1
	residents	3.0	1.1	2.8	1.3	2.6	1.3	2.5	1.3	2.7	1.3	2.7	1.2
	no specialization	3.0	1.2	2.7	1.3	2.2	1.1	2.3	1.3	2.7	1.3	2.5	1.2
	p^‡^	0.001		<0.001		0.005		0.078		0.586		0.006	

*M – mean; SD – standard deviation.

†t-test for independent samples.

‡one-way ANOVA for independent samples.

### Relationship between burnout and workplace stressors

Correlation analysis showed that all examined domains of professional stressors were significantly and positively correlated with all dimensions of burnout (total, personal, work-related, and patient-related burnout; *P* < 0.001). These findings indicated that higher exposure to work stressors was associated with higher levels of burnout (Table 6). The strongest correlations were observed between workplace organization and financial issues and total burnout (r = 0.397), as well as work-related burnout (r = 0.393). The weakest correlations were found between conflict and communication at work and patient-related burnout (r = 0.205).

**Table 6:** Correlations between the professional stressors and burnout dimensions among the health care professionals in FBiH

	Workplace organization and financial issues	Public criticism	Dangers and harmfulness at work	Conflict and communication at work	Shift work	Professional and intellectual demands
Total burnout	0.397*	0.378*	0.335*	0.336*	0.315*	0.345*
Personal burnout	0.363*	0.338*	0.320*	0.344*	0.303*	0.343*
Work-related burnout	0.393*	0.361*	0.305*	0.347*	0.317*	0.338*
Patient-related burnout	0.303*	0.308*	0.273*	0.205*	0.217*	0.247*

*significant at the 0.01 level (2-tailed); Pearson correlation coefficients between the analyzed variables.

The results of the multiple regression analysis used to examine the predictive effects of workplace stressors on overall burnout and individual burnout dimensions are presented in Table 7.

**Table 7:** Multiple regression analysis of work-related stressors as predictors of burnout

	Model 1		Model 2		Model 3		Model 4	
	Total burnout		Personal burnout		Work-related burnout		Patient-related burnout	
	β	95% CI	β	95% CI	β	95% CI	β	95% CI
Workplace organization and financial issues	0.14	−0.45; 4.41	0.05	−1.82; 3.15	0.16	−0.31; 5.39	0.16	−0.44; 5.67
Public criticism	0.14	−0.06; 3.76	0.09	−0.72; 3.16	0.09	−0.89; 3.57	0.19	0.69; 5.46
Dangers and harmfulness at work	0.01	−1.58; 1.84	0.01	−1.60; 1.87	−0.05	−2.79; 1.20	0.07	−0.93; 3.34
Conflict and communication at work	0.12	−0.12; 3.27	0.18	0.68; 4.12	0.16	0.56; 4.53	−0.02	−2.50; 1.74
Shift work	0.02	−1.31; 1.84	0.06	−0.87; 2.34	0.06	−0.95; 2.74	−0.06	−2.92; 1.02
Professional and intellectual demands	0.02	−1.60; 2.26	0.07	−1.00; 2.92	0.02	−2.01; 2.50	−0.01	−2.62; 2.20
	R = 0.406; Adjusted R^2^ = 0.155; *P* < 0.001		R = 0.397; Adjusted R^2^ = 0.148; *P* < 0.001		R = 0.398; Adjusted R^2^ = 0.149; *P* < 0.001		R = 0.312; Adjusted R^2^ = 0.087; *P* < 0.001	

Total burnout: The first model was statistically significant (*P* < 0.001), explaining 15.5% of the variance. However, no individual stressor stood out as a unique predictor. This suggests that overall burnout arises from the combined influence of multiple workplace stressors rather than from any individual factor.

Personal burnout: The second model was also significant (*P* < 0.001), accounting for 14.8% of the variance. Conflicts and communication at work was identified as a significant individual predictor (*P* < 0.01), which indicates that higher levels of stress from interpersonal conflicts and poor communication were associated with increased personal burnout.

Work-related burnout: The third model was also significant (*P* < 0.001), accounting for 14.9% of the variance. Conflicts and communication at work again emerged as a significant predictor (*P* < 0.05).

Patient-related burnout: The fourth model accounted for 8.7% of the variance (*P* < 0.001). Here, public criticism was the only significant predictor (*P* < 0.05), which suggests that negative feedback or criticism from the public primarily affects burnout associated with patient interactions.

## Discussion

Our study found that burnout remained highly prevalent among health care professionals in the FBiH, with 53% of respondents reporting symptoms of overall burnout and 75.7% experiencing at least one dimension of burnout.

Personal burnout was the most common (70.9%), followed by work-related burnout (54.2%) and patient-related burnout (40.1%). This pattern, in which personal and work-related exhaustion outweighs patient-related burnout, suggests that the main source of strain stems from the overall work environment and life stress, while the professional relationship with patients is largely preserved. Very similar results were obtained in our study on burnout during the pandemic in health care workers in the FBiH (23). Similar burnout trends, with lower scores on patient-related burnout compared with other domains, have also been reported in other studies based on the CBI (13,31-33).

Female respondents reported higher levels of personal burnout, which may reflect higher work-life interference among women. Although this may be explained by the majority of the respondents being women, these findings are consistent with earlier reports (34-36).

Patient-related burnout was higher among professionals working in primary health care and among physicians without a specialization, likely due to longer, more direct patient contact and limited resources, consistent with previous findings (23).

All occupational stressors were significantly correlated with burnout across dimensions. Regression analyses further revealed that overall burnout resulted from cumulative stress rather than a single stressor. This supports the understanding of burnout as a multifactorial phenomenon in which individual stressors rarely act in isolation, but rather their effects are summed across multiple domains of work and life stress. However, when we examined specific dimensions, distinct predictors emerged. The primary individual predictors for personal and work-related burnout were conflicts and communication at work, encompassing poor communication and tensions with superiors and colleagues. In contrast, the only significant predictor for patient-related burnout was public criticism, a factor that includes external pressures such as the threat of lawsuits, misinformed patients, conflicts with the patient or family members of the patient, and the challenge of separating professional and private life. In a meta-analysis by Amiri et al, all the examined risk factors were significantly positively related to burnout incidence, with key factors being workplace bullying, high job stress, and poor communication (11). According to a review of predictors associated with burnout among US health care providers, significant predictors of burnout were unsupportive leadership, workload, job autonomy, and poor work-life balance (37). Protective factors included supportive work environments and leadership, adequate staffing, individual resilience, perceived autonomy, and sufficient time spent away from work (11,37).

Although this study highlighted insufficient staffing, administrative burden, work overload, and shift work as the most intense stressors, personal and work-related burnout were most strongly associated with conflictual relationships and poor quality of communication. This finding indicates that interventions should not be focused exclusively on workload, but also on interpersonal relationships and work culture in institutions. Profession-specific differences were also apparent: nurses and technicians experienced greater stress related to occupational hazards, while physicians more often highlighted shift work as a key source of stress, which indicates the need for targeted, profession-specific burnout prevention measures. Similarly, West et al pointed out the need for both individual-focused and organizational strategies for meaningful reduction of burnout (38).

A notable paradox relates to comparing stress perception and burnout levels in terms of age and experience. Specialists and more experienced physicians reported significantly lower levels of stress related to workplace organization, public criticism, and professional demands; yet, they had higher levels of total and work-related burnout. This suggests that while age, experience, professional autonomy, and clinical authority may buffer the immediate perception of stress, they do not necessarily protect against the cumulative toll of long-term responsibility. Our findings diverge from much of the existing literature, which often identifies residents as the most vulnerable group (34,35).

Several potential limitations of this study should be acknowledged. This is a cross-sectional study, and all data are self- reported, which might have introduced bias. The sample may not entirely represent all the HCWs in FBiH, and may be biased toward HCWs experiencing dissatisfaction or burnout, as those without burnout may have been less motivated to respond. Also, factors such as personality traits, coping mechanisms, mental health history, organizational support, or external life stressors were not measured but could influence burnout.

In conclusion, burnout is a concerning, prevalent issue among physicians and medical technicians/nurses in FBiH with patterns similar to those observed during the pandemic. The absence of individual significant predictors in the overall CBI model suggests a high intercorrelation among stressors, which impact general burnout levels collectively rather than individually. Interventions should therefore adopt a holistic, organizational approach, addressing interpersonal dynamics, communication, and work culture, alongside profession-specific stressors.
