# Supplementary material for: The association of burnout with occupational stressors among physicians and medical technicians/nurses in the Federation of Bosnia and Herzegovina: a cross-sectional study
Source: Croat Med J. 2026 Apr;67(2):85–94. doi: 10.3325/cmj.2026.67.85 (PMC13176950; doi:10.3325/cmj.2026.67.85)
Supplement: Supplementary Table 1 [file CroatMedJ_67_s005.pdf]

Supplemental table 1. Average stress ratings of individual stressors

No.	Stressor	M	SD
1	Work overload	2.95	1.42
2	Poor work organization	2.98	1.46
3	Overtime work	2.62	1.42
4	Shift work	2.38	1.36
5	Night work	2.87	1.53
6	On-call (24 hours)	2.96	1.54
7	Pressure of deadlines for completing tasks	2.66	1.41
8	Time limit for examining patients	2.73	1.42
9	Introduction of new technologies	2.20	1.32
10	"Bombing" with new information from the profession	2.27	1.34
11	Lack of adequate permanent education	2.79	1.48
12	Unavailability of necessary literature	2.50	1.41
13	Inadequate material resources for work (financial constraints)	2.88	1.47
14	Inadequate workspace	2.83	1.51
15	Inadequate personal income	2.77	1.47
16	Poor communication with superiors	2.68	1.49
17	Poor communication with colleagues	2.32	1.34
18	Little opportunity for advancement and promotion	2.70	1.50
19	Administrative work	3.03	1.51
20	Insufficient number of employees	3.06	1.55
21	Everyday unforeseen or unplanned situations	2.72	1.44
22	Conflicts with superiors	2.43	1.46
23	Conflicts with colleagues	2.30	1.43
24	Conflicts with other associates	2.21	1.37
25	Conflicts with the patient or family members	2.32	1.39
26	Exposure to inappropriate public criticism	2.60	1.51
27	Threat of lawsuit and litigation	2.55	1.53
28	Inability to separate professional and private work	2.48	1.43
29	24-hour responsibility	2.89	1.53
30	Inadequate expectations from patients and families	2.66	1.45
31	Misinforming patients by the media and other sources	2.85	1.51
32	Dealing with incurable patients	2.72	1.44
33	Fear of exposure to ionizing radiation	2.38	1.38
34	Fear of exposure to inhalation anaesthetics	2.29	1.35
35	Fear of exposure to cytostatics	2.37	1.40
36	Fear of possible infection from sick people	2.29	1.36
37	Fear of possible injury from a sharp object	2.23	1.37

M: mean. SD: standard deviation.
